# Mapping an undergraduate medical education curriculum against national and international palliative care reference learning objectives

**DOI:** 10.1186/s12909-024-05082-1

**Published:** 2024-02-01

**Authors:** Rebekah Murphy, Christopher J. Barnes, Paula D. Enright, Valerie Gratton, Shirley H. Bush

**Affiliations:** 1 Department of Medicine, Division of Palliative Care, University of Ottawa, 451 Smyth Rd, Ottawa, ON K1H 8M5 Canada; 2 Department of Palliative Care, Bruyère Continuing Care, Saint-Vincent Hospital, 60 Cambridge St N, Ottawa, ON K1R 7A5 Canada; 3 Department of Medicine, Queensway Carleton Hospital, 3045 Baseline Rd, Ottawa, ON K2H 8P4 Canada; 4 Ottawa Hospital Research Institute, 1053 Carling Ave, Ottawa, ON K1Y 4E9 Canada; 5 Bruyère Research Institute, 43 Bruyère St, Ottawa, ON K1N 5C8 Canada; 6 Institut du Savoir Montfort, 713 Montréal Rd, Ottawa, ON K1K 0T2 Canada; 7 Department of Family Medicine, Montfort Hospital, 713 Montréal Rd, Ottawa, ON K1K 0T2 Canada

**Keywords:** Palliative care, Curriculum map, Medical education, Undergraduate, Curriculum assessment

## Abstract

**Background:**

The teaching of palliative care competencies is an essential component of undergraduate medical education. There is significant variance in the palliative care content delivered in undergraduate medical curricula, revealing the utility of reference standards to guide curricular development and assessment. To evaluate our university’s undergraduate palliative care teaching, we undertook a curriculum mapping exercise, comparing official learning objectives to the national Educating Future Physicians in Palliative and End-of-Life Care (EFPPEC) and the international Palliative Education Assessment Tool (PEAT) reference objectives.

**Methods:**

Multiple assessors independently compared our university’s UGME learning objectives with EFPPEC and PEAT reference objectives to determine the degree-of-coverage. Visual curriculum maps were created to depict in which part of the curriculum each objective is delivered and by which medical specialty.

**Results:**

Of 122 EFPPEC objectives, 55 (45.1%) were covered fully, 42 (34.4%) were covered partially, and 25 (20.5%) were not covered by university objectives. Of 89 PEAT objectives, 40 (44.9%) were covered fully, 35 (39.3%) were covered partially, and 14 (15.7%) were not covered by university objectives.

**Conclusions:**

The majority of EFPPEC and PEAT reference objectives are fully or partially covered in our university’s undergraduate medical curriculum. Our approach could serve as a guide for others who endeavour to review their universities’ specialty-specific medical education against reference objectives. Future curriculum development should target the elimination of identified gaps and evaluate the attainment of palliative care competencies by medical learners.

**Supplementary Information:**

The online version contains supplementary material available at 10.1186/s12909-024-05082-1.

## Background

Most physicians and medical learners, regardless of specialty, level of training, or geographic location, will care for patients with palliative care needs. The importance of building palliative care competency into early medical training is well-recognized, yet a growing body of world-wide literature highlights ongoing deficits in undergraduate palliative care training [1–7].

The utility of reference palliative care competencies to guide the assessment and reform of palliative care undergraduate medical curricula drove the development of the Palliative Education Assessment Tool (PEAT) in 2000. It was created in the United States as a “flexible self-assessment tool to determine the existence of palliative care education in a wide range of curriculum formats” [8]. Its design, importantly, supports the identification of palliative care content that is delivered outside of specific palliative care teaching sessions [8]. PEAT consists of 89 specific learning objectives categorized into seven major palliative care domains and has been used to assess international curricula [8–12].

In Canada, the need for medical learners to acquire essential palliative care competencies has similarly been emphasized [13, 14]. The Educating Future Physicians in Palliative and End-of-Life Care (EFPPEC) project set Canadian national undergraduate medical education (UGME) competencies in 2008, and a validated update was published in 2018 [15]. The updated EFPPEC consists of 122 objectives that enable the acquisition of 10 overarching palliative care competencies [16].

The original EFPPEC objectives were used in 2009 to develop a palliative care curriculum for the UGME program at our university, the University of Ottawa [17]. The updated EFPPEC competencies/objectives, coupled with an increasing global focus on improving undergraduate palliative care education, compelled us to create curriculum maps of the University of Ottawa’s current UGME intended palliative care content using EFPPEC and PEAT as reference standards to identify priorities for further curriculum development.

The process of curriculum mapping identifies and visually represents when, where, and how educational programs impart specific competencies [18–20]. Curriculum maps can focus on the intended curriculum (official university objectives), the delivered curriculum (what is taught by the educators who are assigned the intended objectives) and/or the learned curriculum (the knowledge and skills learners acquire through the delivered curriculum) [19–21]. Mapping of an intended curriculum can highlight missing objectives so that important curricular content can be added. It may also identify redundant objectives that could be removed to make room for new content. Taught and learned curricula can be mapped as well, which allows targeted interventions to improve teaching effectiveness.

## Methods

### Setting and scope

The 4-year UGME program at the University of Ottawa, Ottawa, Canada consists of two years of preclerkship followed by two years of clerkship. There is one week dedicated to palliative care teaching during the second year of preclerkship, which includes 16 h of didactic and 4 h of small group interactive teaching. While curriculum maps can be presented in various forms with different foci and functionalities, the scope of this project was to examine and display the University of Ottawa’s intended UGME learning objectives using EFPPEC (2018) and PEAT (2000) as reference standards.

### Process (September 2018 – November 2020)

The project lead (RM) reviewed the approximately 6,400 official university UGME objectives (2018 version) and recorded those that appeared to cover any part of an EFPPEC or PEAT reference objective on a Microsoft Excel® spreadsheet [22]. The project lead was purposely overly-inclusive during this step to ensure no potential matches were excluded. Data extraction for university objectives included which EFPPEC and/or PEAT objective(s) they potentially addressed (thus creating university-reference objective pairings), the UGME year, the unit/rotation, and by which medical specialty they are taught.

**Fig. 1:**
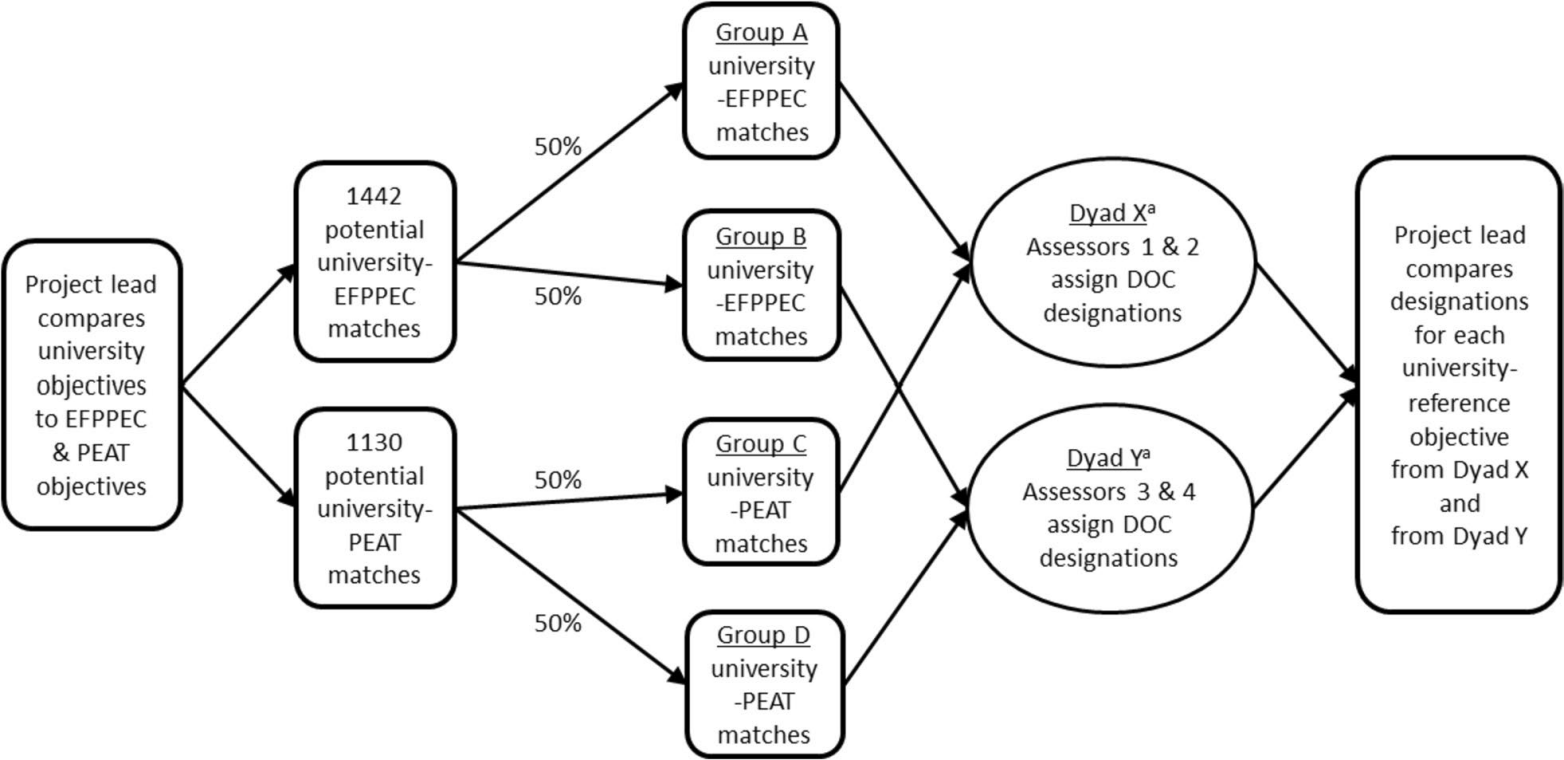
Flowchart depicting the process of dividing university-reference objective pairings and assigning degree-of-coverage designations *Legend:* EFPPEC: Educating Future Physicians in Palliative and End-of-Life Care reference objectives. PEAT: Palliative Education Assessment Tool reference objectives. DOC: Degree-of-coverage. Groups A-D are groupings of potential university-reference objective pairings. ^a^Note: The assessors in each dyad reviewed the same university-reference objective pairings, but they reviewed and assigned degree-of-coverage designations independently of each other

Potential university-reference objective pairings were divided into four groups and each group was assigned to assessor dyads (VG, SB, PE, CB). (See Fig. 1.) After piloting and discussing 50 university-reference objective pairings as a team to minimize inter-assessor variability, assessors independently ascribed each of their assigned objective pairings to one of three defined degree-of-coverage designations: ‘fully covered’ (university objective covered all of the reference objective); ‘partially covered’ (university objective covered any part, but not all, of the reference objective); and ‘not covered’ (university objective did not cover any of the reference objective). The project lead then compared the two degree-of-coverage designations assigned by the assessor dyads for every university-reference objective pairing, and for pairings with conflicting designations, made the final determination.

Finally, curriculum maps were created on Microsoft Excel® spreadsheets, one for the university-EFPPEC and a second for the university-PEAT pairings, to depict the degree to which the university objectives cover the reference objectives. The maps also display the year/unit and by which medical specialty the reference objectives are covered.

### Ethics waiver

As this project was a quality initiative with no human subjects, the applicable research ethics boards granted exemptions.

## Results

Of the 122 EFPPEC reference objectives, 55 (45.1%) were fully covered by university objectives, with 19 (15.6%) of these using identical wording. Forty-two (34.4%) EFPPEC objectives were partially covered, while 25 (20.5%) EFPPEC objectives were not covered by university objectives. Of the 89 PEAT reference objectives, 40 (44.9%) were fully covered, 35 (39.3%) were partially covered, and 14 (15.7%) were not covered by university objectives. In general, coverage of both EFPPEC and PEAT objectives occurred more frequently in year 2, 3, and 4 of the university UGME curriculum. See Tables 1 and 2 for further details.

**Table 1:** Number of EFPPEC^a^ objectives fully, partially, and not covered by university objectives in overarching competencies

EFPPEC^a^ overarching competency (N reference objectives)	Fully covered N (%)	Partially covered N (%)	Not covered N (%)
1. Palliative approach to care (4)	1 (25%)	0 (0%)	3 (75%)
2. Pain & symptom management (34)	16 (47.1%)	12 (35.3%)	6 (17.6%)
3. End of life care (6)	3 (50%)	2 (33.3%)	1 (16.7%)
4. Pediatric palliative care (6)	4 (66.7%)	0 (0%)	2 (33.3%)
5. Psychosocial & spiritual care (15)	7 (46.7%)	4 (26.7%)	4 (26.7%)
6. Bioethical & legal end-of-life decision-making (25)	12 (48%)	12 (48%)	1 (4%)
7. Effective communication (15)	5 (33.3%)	6 (40%)	4 (26.7%)
8. Interprofessional collaboration (9)	4 (44.4%)	4 (44.4%)	1 (11.1%)
9. Multidimensional sources of suffering (5)	2 (40%)	1 (20%)	2 (40%)
10. Self-awareness & self-care (3)	1 (33.3%)	1 (33.3%)	1 (33.3%)

^a^*EFPPEC* Educating future physicians in palliative and end-of-life care reference objectives

**Table 2:** Number of PEAT^a^ objectives fully, partially, and not covered by university objectives in PEAT domains

PEAT^a^ domain (N reference objectives)	Fully covered N (%)	Partially covered N (%)	Not covered N (%)
I. Palliative Medicine (6)	0 (0%)	2 (33.3%)	4 (66.7%)
II. Pain (12)	5 (41.7%)	6 (50%)	1 (8.3%)
III. Neuropsychological Symptoms (11)	8 (72.7%)	3 (27.3%)	0 (0%)
IV. Other Symptoms (10)	5 (50%)	5 (50%)	0 (0%)
V. Ethics & the Law (20)	11 (55%)	5 (25%)	4 (20%)
VI. Patient/Family/Nonclinical Caregiver Perspectives on End-of-life Care (12)	3 (25%)	7 (58.3%)	2 (16.7%)
VII. Clinical Communication Skills (18)	8 (44.4%)	7 (38.9%)	3 (16.7%)

^a^*PEAT* Palliative education assessment tool reference objectives

**Fig. 2:**
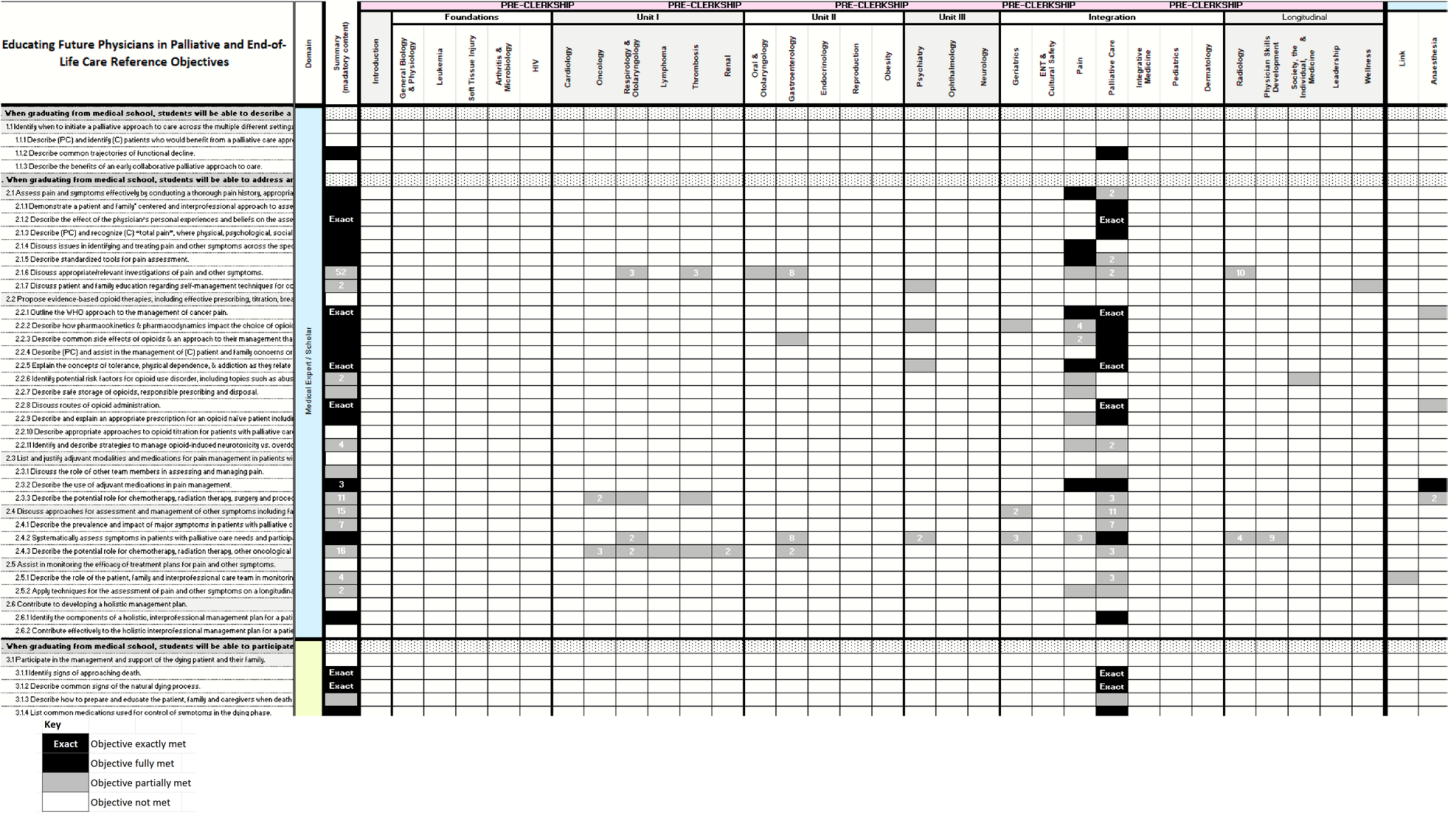
Portion of EFPPEC Curriculum Map *Legend:* EFPPEC: Educating Future Physicians in Palliative and End-of-Life Care reference objectives. First column from the left contains EFPPEC reference objectives. Second column from the left contains EFPPEC domains. Third column from the left shows if EFPPEC reference objective was covered anywhere in the four-year undergraduate medical education curriculum. Top row shows whether EFPPEC reference objective was covered in preclerkship or clerkship. Second from the top row shows in which unit EFPPEC reference objective was covered. Third row from the top shows by which specialty EFPPEC reference objective was covered. Numbers in shaded boxes denote that there are multiple university objectives that cover the reference objective to the degree denoted by the shade of the box (and indicate how many objectives of that degree-of-coverage). See Additional file 1: Table 1, for full EFPPEC Curriculum Map

**Fig. 3:**
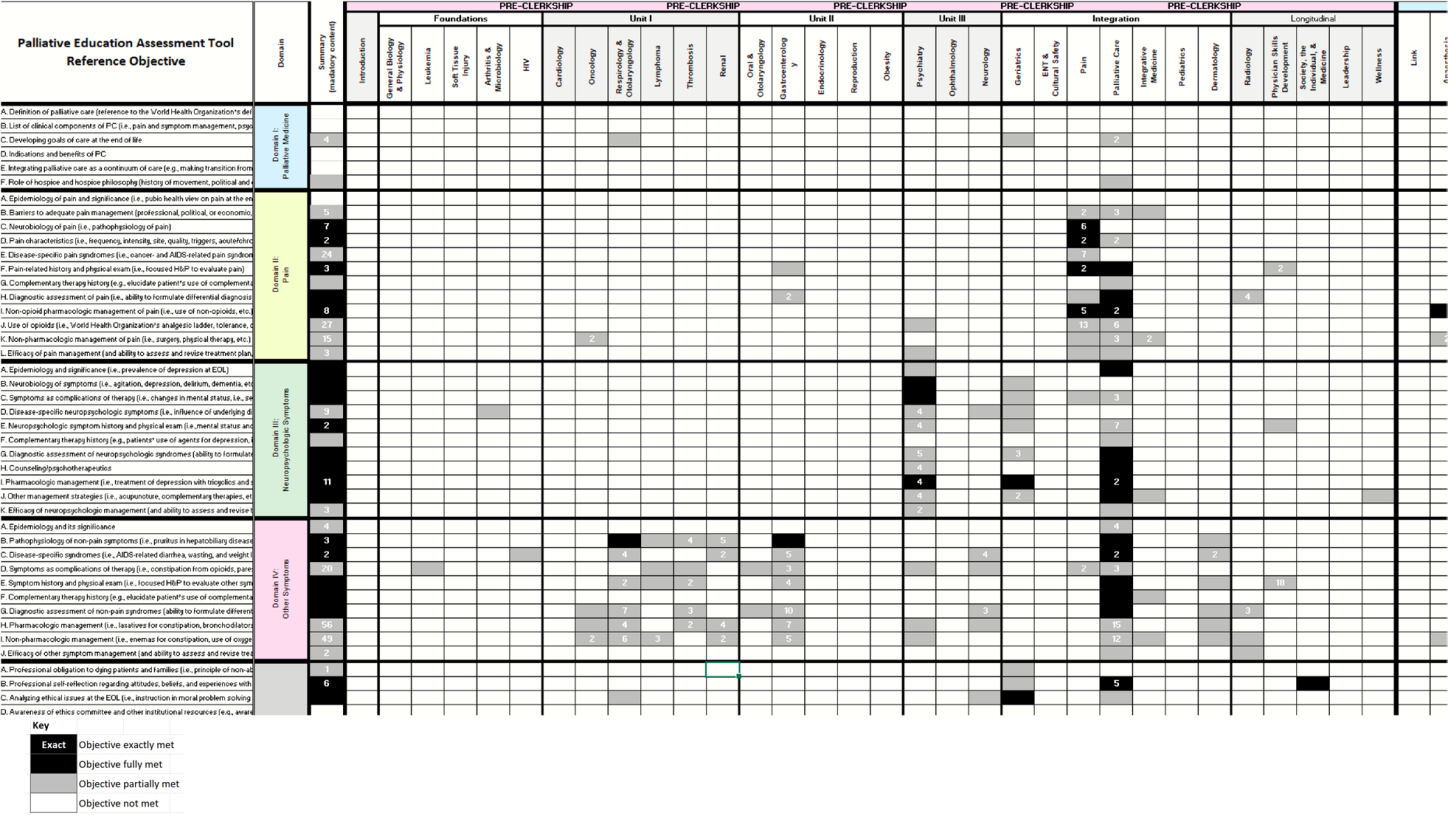
Portion of PEAT Curriculum Map *Legend:* PEAT: Palliative Education Assessment Tool reference objectives. First column from the left contains PEAT reference objectives. Second column from the left contains PEAT domains. Third column from the left shows if a PEAT reference objective was covered anywhere in the four-year undergraduate medical education curriculum. Top row shows whether PEAT reference objective was covered in preclerkship or clerkship. Second from the top row shows in which unit PEAT reference objective was covered. Third row from the top shows by which specialty PEAT reference objective was covered. Numbers in shaded boxes denote that there are multiple university objectives that cover the reference objective to the degree denoted by the shade of the box (and indicate how many objectives of that degree-of-coverage). See Additional file 1: Table 2, for full PEAT Curriculum Map

An additional Microsoft Excel® file provides the full EFPPEC and PEAT curriculum maps in separate spreadsheet tabs. Figures 2 and 3 show select higher resolution portions of the EFPPEC and PEAT curriculum maps, respectively.

## Discussion

Our curriculum mapping project revealed that the majority of EFPPEC and PEAT reference objectives are currently fully or partially covered in our university’s curriculum. In particular, there are relatively few EFPPEC objectives in the “Bioethical & legal end-of-life decision-making” overarching competency and PEAT objectives in the ‘symptom’ domains (II, III, IV) that are not covered. However, the relatively high percentage of EFPPEC reference objectives in the “Palliative approach to care” overarching competency that are not covered compared to the other overarching competencies is also a noteworthy observation. Similarly, there is a relatively high percentage of PEAT reference objectives in the “Palliative Medicine” domain that are not covered compared to the other domains. The reason for this is unclear, although it may be exaggerated by the relatively few reference objectives in those categories. Another potential explanation is that both of those categories include the most basic information about palliative care, such as the definition and benefits, rather than competencies required to practice palliative care. It is possible that those reference objectives are naturally covered in the university’s delivered curriculum (what is actually taught) without explicitly including them in the intended curriculum (official written objectives). Future assessment of the delivered and learned curricula would make for an interesting comparison and could further inform curriculum improvement.

To our knowledge, this is the first curriculum mapping project that assesses a university’s UGME curriculum separately against both a national and an international palliative care reference standard. At the time this manuscript was being written, we were unable to find any other published curriculum assessment projects that used EFPPEC as a reference. There are several other published curriculum assessment studies that have referenced PEAT [9–12], but most of their aims and methods are quite dissimilar to our project. For example, Kim et al [9] assessed end of life care curricula by surveying directors or faculty members of 27 Korean medical schools, while Schiessl et al [10] reviewed 17 international UGME curricula identified by a literature search, with both studies reporting the proportion of their respective curricula that covered each PEAT objective and domain. The different modes of data collection and output limit the utility of comparing results of our project to theirs.

The general aim of the Lehto et al [12] study, which assessed their university’s curriculum against two international palliative care references, the European Association for Palliative Care (EAPC) recommendations and PEAT, is most similar to that of our project. However, their methods indicate that the PEAT domains were integrated into the six sections described by EAPC prior to curriculum assessment. Like our finding, they have a high degree of compliance between their university’s curriculum and the reference standards they used, but the dissimilarity in type and format of their results limits further comparison. The example curriculum map display reported by Wood et al [11] is reasonably analogous to our curriculum map display, but because their main emphasis is on multi-school curriculum development following the mapping process, the utility of comparison is similarly ambiguous. The findings of their study, however, may be relevant to future use of our findings for palliative care curriculum optimization.

The 25 EFPPEC and 14 PEAT objectives that are not yet covered by our university’s UGME curriculum solicit consideration of how to best incorporate them. Several studies emphasize the challenge of adding more palliative care content to already crowded UGME curricula [11, 23, 24]. Though much of our university’s palliative care content is delivered in a designated palliative care teaching block during the second year of preclerkship, we found objectives that cover EFPPEC and PEAT objectives throughout the four-year curriculum and taught by numerous non-palliative care medical specialties. This phenomenon is also noted in the aforementioned Lehto et al. study [12] and in a longitudinal perspective study on UGME palliative care training in Germany by Ilse et al. [25] Our finding invites advancement of the reported benefits of having palliative care content woven throughout the entire UGME curriculum, such as longitudinal exposure mirroring the ubiquitous application of palliative care across the spectrum of medicine and promoting learners’ sustained awareness of its value [11, 26].

EFPPEC [15] is the national UGME palliative care reference for the University of Ottawa, making it an obvious reference standard for comparison. PEAT [8] was a prime international reference with which to assess our university’s curriculum because it is designed to assess all UGME teaching across a 4-year curriculum, not only dedicated palliative care content. Other reference standards for UGME palliative care teaching are available, including the Association of Palliative Medicine (APM) for Great Britain and Ireland’s syllabus [27] and the Netherlands’ Palliative care, Alliance, Sharing, Educational tools for MEdical student Competencies (Pasemeco) [6], but the APM syllabus has been described as too lengthy to embed in already overcrowded UGME curricula [23] and Pasemeco was only published in 2020 when this project’s data extraction was nearing completion. Ultimately, the theoretical utility of comparing our university’s curriculum with the APM syllabus and Pasemeco is unclear because medical training is structured quite differently in the United Kingdom [28, 29] and the Netherlands [30, 31] compared with North America [32, 33].

Our curriculum mapping method could be used as a model for other universities that are undertaking UGME palliative care curriculum development, or as a guide for other specialties that are interested in evaluating their curriculum compared to reference standards. While the comprehensive method of data extraction was time-consuming, we gleaned detailed data that allowed in-depth comparison and we were able to save considerable cost for this non-funded project that would have been required for curriculum mapping software.

### Strengths and Limitations

A project strength is that the granularity of our data facilitated recognition of palliative care content taught outside of the core palliative care teaching sessions and highlighted redundant coverage of some reference objectives. The data collection format, in which each university-reference objective pairing is recorded in detail, also allows future determination of whether reference objectives shown on the map as partially covered by more than one university objective may actually be fully covered by the “sum” of multiple university objectives. A major challenge of teaching comprehensive palliative care in UGME is the lack of available curriculum hours. Thus, eliminating redundant university objectives, identifying reference objectives that are fully covered by the combination of multiple university objectives, and recognizing existing palliative care content in teaching sessions by non-palliative specialties, as supported by our mapping method, could aid in overcoming this challenge.

A project limitation is the subjective nature of assigning degree-of-coverage designations to university-reference objective pairings. For example, the university objective, “Recognize the goals of end-of-life decision making” does not completely cover the reference objective, “Developing goals of care at the end of life.” The verbs ‘recognize’ and ‘develop’ require different levels of skill, conferring complexity to degree-of-coverage assignment. Additionally, differences between the wording used in EFPPEC and PEAT reference objectives and local terminology may have influenced university objectives’ degree-of-coverage assignments. We attempted to mitigate subjectivity with the pilot phase to minimize inter-assessor variability.

A caveat is that this curriculum mapping project assessed our university’s intended curriculum. There is likely to be degradation between an ideal intended curriculum and its resultant delivered and learned curricula for many reasons, some of which include particular content selection, teaching methodology, and powerful modulation of the learner experience by informal and hidden curricula [20, 21]. Thus, assessment of the delivered and learned curricula are also important measures to consider. At our university, educators must reference the official objective number from the intended curriculum when creating exam questions for learner evaluation. As such, we first needed to determine if and which university objectives covered the EFPPEC & PEAT objectives before we could eventually employ the system that is already in place for evaluating the learned curriculum. The rigorous de novo process of mapping the intended curriculum would have been prohibitively complex if the delivered and/or learned curricula had been simultaneously assessed.

## Conclusions

A curriculum mapping exercise can be employed to evaluate a UGME palliative care curriculum and may enable educators to optimize the intended curriculum. Our approach could be used as a curriculum assessment prototype for other universities or programs, with adjustments based on program-specific goals and resources. Future opportunities stemming from this project include optimization of our university’s UGME palliative care intended curriculum, as well as the development of studies to examine delivered and attained palliative care competencies.

### Supplementary Information

**Additional file 1.** 

## Data Availability

Additional file 1 is a Microsoft Excel® file with 2 tabs that contain the full EFPPEC curriculum map spreadsheet in the first tab and the full PEAT curriculum map spreadsheet in the second tab. The Microsoft Excel® data collection spreadsheet that catalogues the University of Ottawa objectives is a large file with university-specific information. As it is unlikely to be widely applicable, it has not been included in this publication. It can be made available from the corresponding author on reasonable request.

## Abbreviations

PEAT: Palliative education assessment tool
EFPPEC: Educating future physicians in palliative and end-of-life care
UGME: Undergraduate medical education
DOC: Degree-of-coverage
EAPC: European association for palliative care
APM: Association of palliative medicine
Pasemeco: Palliative care, alliance, sharing, educational tools for medical student competencies
